# Regions of intensification of extreme snowfall under future warming

**DOI:** 10.1038/s41598-021-95979-4

**Published:** 2021-08-17

**Authors:** Lennart Quante, Sven N. Willner, Robin Middelanis, Anders Levermann

**Affiliations:** 1 Potsdam Institute for Climate Impact Research, Telegrafenberg A56, Potsdam, Germany; 2 Potsdam University, Karl-Liebknecht Str. 24, Potsdam, Germany; 3 Columbia University, LDEO, Palisades, NY USA

**Keywords:** Atmospheric science, Climate change

## Abstract

Due to climate change the frequency and character of precipitation are changing as the hydrological cycle intensifies. With regards to snowfall, global warming has two opposing influences; increasing humidity enables intense snowfall, whereas higher temperatures decrease the likelihood of snowfall. Here we show an intensification of extreme snowfall across large areas of the Northern Hemisphere under future warming. This is robust across an ensemble of global climate models when they are bias-corrected with observational data. While mean daily snowfall decreases, both the 99th and the 99.9th percentiles of daily snowfall increase in many regions in the next decades, especially for Northern America and Asia. Additionally, the average intensity of snowfall events exceeding these percentiles as experienced historically increases in many regions. This is likely to pose a challenge to municipalities in mid to high latitudes. Overall, extreme snowfall events are likely to become an increasingly important impact of climate change in the next decades, even if they will become rarer, but not necessarily less intense, in the second half of the century.

Global warming caused by persisting greenhouse gas emissions^1^ is expected to cause an increasing number of extreme weather events^2,3^. The intensification of precipitation events^4^ is one of the main consequences of global warming. One of the main driving factors for this is the increase of global mean temperature of around 1 °C over the last century and the projected future increase of a similar magnitude in the next decades. Rising temperatures lead to higher evaporation and thus enable more global precipitation. Since higher temperatures also increase atmospheric water vapour, this may lead to more intense extreme rainfall events^1^.

Snowfall and its extremes are a special case of precipitation, since the intensification of the hydrological cycle allows for potentially more snowfall, as long as temperatures remain sufficiently cold for snowfall to occur. These opposing forces lead to a contrast between a substantial decrease of mean snowfall and a much less pronounced decrease of extreme snow events^5^. These results based on empirical percentiles and generalised extreme value distribution analysis emerge very pronounced under a high emissions scenario (RCP8.5). O’Gorman^5^ thereby presents a physical theory complementing these findings to show that snowfall extremes occur close to an optimal temperature $T_m$ and thus the change of extreme events related to warming is argued to be differing from the decrease of mean snowfall. A regional comparison^6^ for RCP8.5 shows a general decrease in daily snowfall events in most regions, with exceptions in regions with sufficiently cold climate even under global warming. Reductions of snowfall also cause reduced mountain snow pack under RCP8.5 in North-Western America, leading up to a possible disruption of agriculture due to lack of predictability of melt water occurrence^7^. Further, down-scaled regional climate models show a decreasing size and frequency of snow storms in eastern North America^8^ under RCP8.5.

Even with medium emissions (RCP4.5) a reduction of annual mean snowfall is observed with increases in high latitude regions^9^. And also for a strong CO$_2$ doubling experiment a weaker decrease of wind-driven heavy snowfall events is found when compared to the decrease of mean snowfall^10^. Adding to these model-based analyses of future snowfall, snow mass shows continental contrasts. North America displays decreasing trends of snow mass, while for Eurasia no trend is found using satellite data for the recent past (1980–2018)^11^.

For the latest round of the Climate Model Intercomparison Project, CMIP6, it has been shown that, for a fixed snow threshold and conversion ratio between liquid and solid precipitation, there is an increase in the occurrence of high snowfall events that can be attributed to anthropogenic greenhouse gas emissions in most parts of Asia, North America, and Greenland. By contrast, decreases of intense snowfall days are described for the remaining regions under the stronger warming of SSP5-RCP8.5 compared to SSP1-RCP2.6^12^. Moreover, recently a linear relationship between increasing global surface air temperature and decreasing spring snow cover has been shown by comparing recent global climate model results from CMIP6 to historical data^13^.

Despite the dire long-term prospects of snowfall under global warming in mid-latitudes, extreme snow events remain a major damaging category of extreme weather events, especially in the Northern Hemisphere. Here, snow and winter storms have caused 21.6bn USD of insured losses (in prices of 2000) in the United states from 1949 to 2000, accounting for roughly $4\%$ of all storm induced insured losses^14^. Such damage due to extreme snowfall increased in the United States during the second half of the twentieth century^14^. However, this effect arises primarily because of a growing population and increasing value of assets at risk.

Here, we show a regionally diverging intensification of extreme snowfall events compared to the historical climate until the end of the century. First, we find that, under a strong global warming scenario (SSP5-RCP8.5), extreme percentiles increase for already snow-prone regions also in mid-latitudes. In contrast to this, mean daily snowfall decreases in most areas of the Northern Hemisphere except for high latitudes.

Second, we show that extreme snowfall intensifies at least until the middle of the century as indicated by an increase of the 99.9th percentile of daily snowfall (commonly measured in kg/(m$^2$s)) as well as of the expected magnitude of extreme events exceeding the historic 99.9th percentile. This is pronounced for high-latitude regions, which face intensifying extreme snow events throughout the century. Mid-latitude regions like Western Europe show increasing expected magnitude of extreme events, i.e. the average of events exceeding the historical 99.9th percentile increases. While we observe a decreasing frequency of extreme snowfall events towards the end of the century, this analysis of the most extreme snowfall events indicates that the remaining events might be more extreme than historical experienced (Fig. 1).

This study is based on most up-to-date climate projections from the Sixth Coupled Model Intercomparison Project (CMIP6)^15^. By contrast, CMIP5 data has been shown to underestimate extreme snowfall events and to overestimate average snowfall when compared to observations^16^. Thus, we here use an ensemble of bias-corrected model output^17,18^ from the ISIMIP project with improved representation of extremes. This bias-correction combines a parametric quantile mapping approach to adjust biases in all quantiles of a distribution and to preserve trends in the individual quantiles as described in detail in^17,19^. Using an ensemble of ten model outputs, we compare extreme daily snowfall percentiles of the Northern Hemisphere land mass above 40$^{\circ }$ N for a historical climate (1851–1920) to a strong global warming scenario (SSP5-RCP8.5; we further provide analyses of SSP1-RCP2.6 and SSP3-RCP7.0 scenarios in the supplement). Furthermore, we introduce the measure of *expected extreme magnitude (EEM)* inspired by the financial risk measure of conditional value at risk^20–22^. We define expected extreme magnitude as the mean of all events above a certain percentile from the historical baseline. Thus, it reflects changes in the tail of the distribution, i.e., for the intensity of extreme events. We overall contribute to the discussion of appropriate measures for extreme precipitation^23^ and suggest the expected extreme magnitude to enable a substantial extension of the analysis of extreme event risk in general. Details are given in the methods, Eq. (2).

Since one of the main biases of global circulation models consists in the underestimation of extreme precipitation events^16^, we are confident that using the bias-corrected data yields a more realistic representation of the extreme percentiles we analyse in our study. As shown in Figs. S7–S11, CMIP model data without bias correction from the GCM MPI ESM1-2-HR leads to quite consistent trends in the mean and EEM, while the single model percentile is more noisy and an increase can not be observed as clearly as for the bias corrected data.

## Global changes of daily snowfall

**Figure 1:**
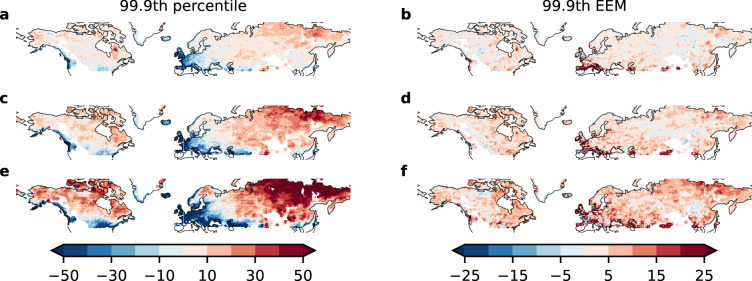
Intensification of extreme daily snowfall throughout the century for high-latitudes, decreasing percentiles, particularly in the second half of the century for mid-latitudes, especially Western Europe. Values are relative to the historical baseline (1851–1920). Relative change (in %) of (**a**,**c**,**e**) 99.9th percentile, and (**b**,**d**,**f**) 99.9th expected extreme magnitude; (**a**,**b**) 2021–2030, (**c,d**) 2051–2060, (**e**,**f**) 2091–2100. Maps created using the cartopy 0.17^24^ library based on GSHHG shapes^25^.

We find that, for the first half of the 21st century, the 99.9th percentile of daily snowfall, i.e., the largest daily snowfall in 1000 days, increases for large areas of the Northern Hemisphere by 10–20 percentage points until the decade of 2051–2060, while some areas, e.g. in Western Europe, show decreasing extreme percentiles (all percentages are relative to the historical baseline (1851–1920) values, Fig. 1, first column). Towards the end of the century (2091–2100), this trend diverges into sharply decreasing 99.9th percentiles for lower latitudes like Western Europe and parts of North America (up to $\sim -50\%$ points), and further increasing percentiles (more than $\sim 30\%$ points) in high latitudes. These trends are well grounded in the model ensemble as shown in Fig. S4.

Substantiating these trends, the expected extreme magnitude, i.e., the average of daily snowfall events exceeding the historical 99.9th percentile (for details see the methods, Eq. (2)), increases by 5–10% points until the middle of the century (Fig. 1, second column). This indicates the strengthening of extreme snowfall events. The continued increase until the end of the century to 10–15% points of the baseline level shows that even with rarer extreme events as indicated by decreasing percentiles, the remaining extreme snowfall events are projected to intensify compared to the historical baseline. These findings are not as robust with respect to model agreement as the analysis of the percentile, due to the inherent high uncertainties in tail risk analysis. Nonetheless, the overall trends are still supported by acceptable model agreement (Fig. S5). We advise caution interpreting the results for regions with an observed strong decrease of the percentile like Western Europe.

**Figure 2:**
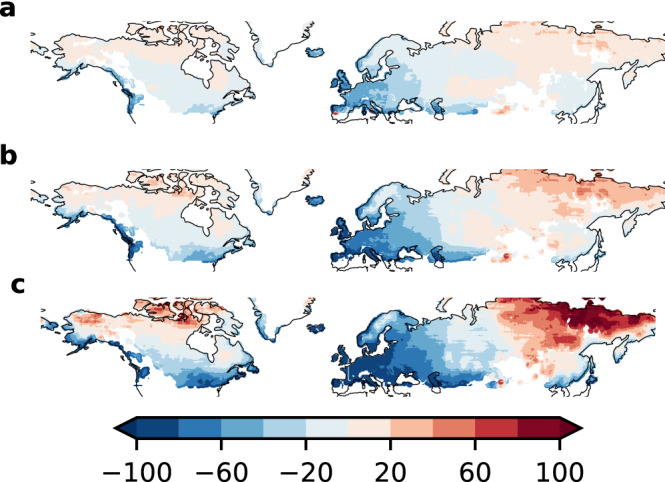
Strong increase in mean daily snowfall in high latitudes of North America and North-East Asia, decreases in mid-latitudes of North America and Western Eurasia relative to historical baseline (1851–1920), (0% points = baseline) (**a**) 2021–2030, (**b**) 2051–2060, (**c**) 2091–2100. Maps created using the cartopy 0.17^24^ library based on GSHHG shapes^25^.

In contrast to this increase in extreme snowfall statistics, the mean daily snowfall diverges already in the near future. While snow-prone regions in high latitudes exhibit an increase of mean daily snowfall by 20% points until the middle of the century, we observe a sharp decrease for lower latitudes ($\sim -20$% points; Fig. 2). These trends continue until the end of the century, yielding decreases of up to 80% points in large parts of Europe and parts of North America, while high-latitude regions like Siberia show a similar increase of around at least 50% points. Again, these trends are well grounded in the model ensemble as shown in Fig. S3.

Possibly due to the improved representation of extreme events in our bias-corrected data^17,18^, we find that the contrast between mean and extreme snowfall might be stronger than discussed previously^5,6^. This is supported by the 99th percentile statistics (Fig. S2). Here, the contrast between higher and lower latitudes is already evident in the middle of the century, with strong decreases in parts of Europe and North America. Nonetheless, the expected extreme magnitude increases by at least roughly 5% points, in support of our claim that extreme snowfall events become more intense, even if their frequency declines as indicated by decreasing percentiles .

**Figure 3:**
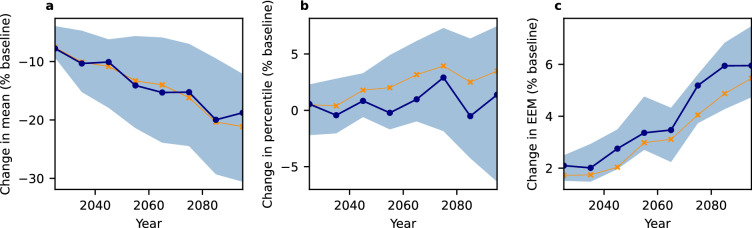
Contrasting global trends of mean daily snowfall and extreme snowfall measures (elevation below 1000 m, decadal statistics, Northern Hemisphere north of 40 °N, SSP5-RCP8.5). All values are area-weighted and relative to the baseline (1851–1920) climate. (**a**) Mean, (**b**) 99.9th percentile, (**c**) expected extreme magnitude above the 99.9th baseline percentile. Blue line shows the model ensemble median, shaded areas denote the likely range (16.7th to 83.3rd percentiles). Orange line shows statistics for all ten models combined into one time series ensemble.

In Fig. 3, we show the area-averaged model ensemble projections for an elevation below 1000 m as high-elevation areas show generally differing snow patterns. While global trends are dampened in comparison to the most volatile regions in Fig. 1, the described divergence between non-decreasing percentiles as well as slightly increasing expected extreme magnitude of daily snowfall and decreasing mean snowfall remains. We observe a decrease of the global mean daily snowfall by almost $20$% points until the end of the century, contrasting a stagnating 99.9th percentile with no clear trend. Some intensification of extreme snowfall events is shown by the increase of 99.9th expected extreme magnitude of daily snowfall by around 4% points. While these trends are heterogeneous between regions, they are still observable in averages for the Northern Hemisphere north of 40°N with narrow likely ranges (Fig. 3, 16.6th to 83.3rd percentiles) in our ensemble of ten bias-corrected climate models^17,18^. This indicates that the trends observed in the simulations are well grounded in our ensemble.

**Figure 4:**
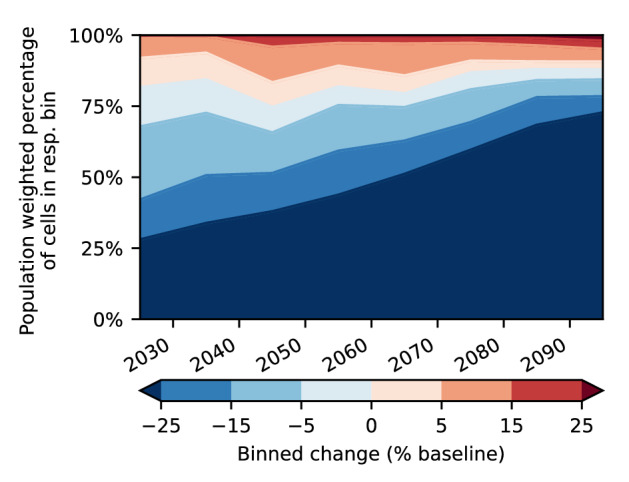
Population exposed to strongly intensifying and decreasing snowfall events grows in the next decades before a majority of population is experiencing decreasing extreme events at the end of the century. Global population weighted trend of 99.9th percentile (elevation below 1000 m, decadal statistics, Northern Hemisphere north of 40 °N, SSP5-RCP8.5). Binned according to change relative to the baseline (1851–1920) climate. Coloured area represents the population weighted percentage of cells in the respective bin. Population is fixed to a 2020 estimate.

To evaluate the possible impacts of this intensification on damages suffered by humans, we analyse the share of the population exposed to increasing or decreasing percentiles of daily snowfall sorted by bins. Until the middle of the century, the population exposed to a strongly increasing 99.9th percentile of daily snowfall (more than 5 percentage points compared to baseline levels) grows slightly by about 10 percentage points, while the population experiencing very strongly (more than 15 percentage points compared to baseline levels) decreasing snowfall events grows by about 10%. Due to the concentration of intensifying daily snowfall events in higher latitudes, this trend is continued till the end of the century and the population experiencing decreasing extreme snowfall grows up to 85% (Fig. 4). Nonetheless, the population weighting shows a still considerable intensification of extreme snowfall in the coming decades, almost doubling the amount of people exposed to strongly intensifying extreme snowfall events, while in the second half of the century decreasing extreme snowfall events will be experienced by an increasing majority of the population. Complementing area weighted analysis is included in the supplement (Fig. S12), showing continuous increase of the area where extreme percentiles intensify very strongly (more than 15 percentage points compared to baseline levels) until the end of the century.

### Regional divergence due to temperature shifts

Since we observe not only a divergence between mean and extreme snowfall but also between different regions, we present some more details on three selected regions: the east coast of North America (1), Western Europe (2), and Northern Asia (3) (Fig. S1)—note that only land cells are considered.

**Figure 5:**
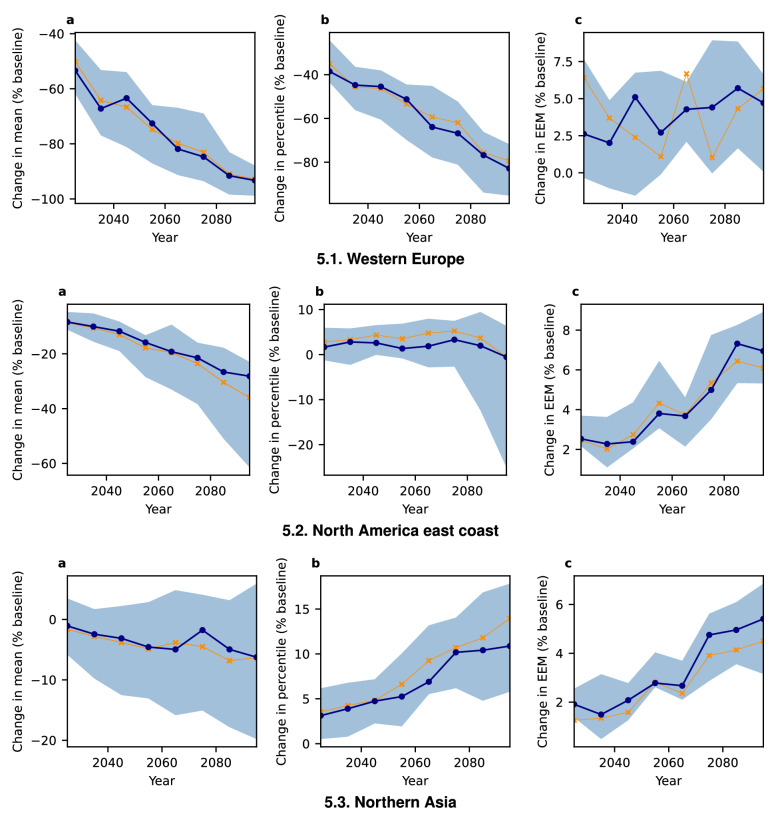
Regional differences of changes in daily snowfall statistics (elevation below 1000 m, decadal statistics, SSP5-RCP8.5). All values are relative to the baseline (1851–1920) climate. (**a**) Mean, (**b**) 99.9th percentile, (**c**) expected extreme magnitude above the 99.9th baseline percentile. Blue line shows the model ensemble median, shaded areas denote the likely range (16.7th to 83.3rd percentiles). Orange line shows statistics for all ten models combined into one time series ensemble.

As shown in Fig. 5, these regions’ snowfall diverges in its response to global warming. While Western Europe exhibits the sharpest decline in average mean snowfall ($\sim -90$% points) and 99.9th percentile ($\sim -80$% points), the few remaining extreme events do not decrease in intensity as shown by the very uncertain, but slight intensification trend ($\sim +5$% points) . The east coast of North America exhibits a much less pronounced decrease of mean snowfall ($\sim -30$% points), while the average percentile remains almost constant. The expected extreme magnitude increases slightly($\sim 5$% points). Snowfall in the higher latitude region of Northern Asia is intensifying with regards to all extreme measures. By contrast, the mean snowfall decreases slightly ($\sim -5$% points). While percentiles of extreme snowfall increase ($\sim 10$% points), the average extreme events show a small increase ($\sim +6$% points). Due to the large magnitude of change, the ensemble does exhibit relatively large uncertainty, but for mean snowfall and the general trends regarding percentiles and expected extreme magnitude, most models show good agreement (Figs. S3–S5). Similar, but slightly weaker trends can be observed for SSP3-RCP7.0 (Fig. S16), whereas SSP1-RCP2.6 induces no clear trend (Fig. S21).

**Figure 6:**
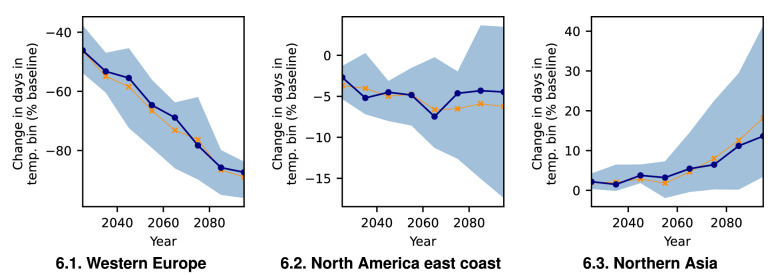
Regional differences in the trend of number of days with surface temperature between ${-2.5}$ $^{\circ }$C and ${-1.5}$ $^{\circ }$C (elevation below 1000 m, decadal statistics, SSP5-RCP8.5). All values are relative to the baseline (1851–1920) climate. Blue line shows the model ensemble median, shaded areas denote the likely range (16.7th to 83.3rd percentiles). Orange line shows statistics for all ten models combined into one time series ensemble.

We find evidence for the existence of an optimal temperature $T_m$ for extreme snowfall in agreement with previous studies on earlier climate projections^5^. For that, we contrast the change of days with surface temperatures below freezing point, respectively, in the bin of $[-2.5\,{^{\circ } \text {C}},-1.5\, {^{\circ }\text {C}}]$ around the proposed^5^ $T_m=-2\,{^{\circ }}$C. We thereby show differences between the considered regions (Fig. 6). While for Western Europe the number of days in the range of $T_m$ is reduced from $\sim 45$% of the historical baseline level in 2020 to $\sim 10$% in the decade 2091–2100 , for the North American east coast these days remain at about $\sim 95$% of the baseline level. For Northern Asia we even observe a small increase to $\sim 110$% in these extreme snowfall prone days. In contrast to this, freezing days in Western Europe are reduced from $\sim 50$% baseline level to $\sim 10$%, the North American east coast shows a much less pronounced reduction from $\sim 90$ to $\sim 60$%, while in Northern Asia freezing days only recede from $\sim 90$ to $\sim 65$% (Fig. S23). Similarly, slightly weaker trends can be observed for SSP3-RCP7.0 (Fig. S17), whereas SSP1-RCP2.6 causes no clear trend (Fig. S22).

We thus conclude that the increase of temperatures renders snowfall so much less likely in Western Europe, that the already observed^26^ and predicted^3,4,27^ intensification of the hydrological cycle does not result in increasing frequency of extreme snowfall events—the increased overall precipitation is not realised as snowfall but as rain. Nonetheless, it leads to a strengthening of the remaining events as indicated by the slight increase in expected extreme magnitude. By contrast, for the east coast of North America as well as Northern Asia, sufficient temperature conditions remain for extreme snowfall to occur. Thus, for these regions we observe intensifying extreme events. On the global scale, we observe an almost steady number of days in the optimal temperature bin with a considerable model spread, whereas freezing days decrease (Fig. S24). These findings indicate that global warming decreases the number of days with sufficiently cold temperatures for snow, while days close to the theorised optimal temperature^5^ of $\sim -2\,{^{\circ }}$C reduce not as much in regions with intensifying extreme snowfall.

## Discussion

In this study, we show that daily extreme snowfall events are projected to intensify in many regions in the latest climate model projections. We introduce the measure of expected extreme magnitude of snowfall events and show increasing tail risk of daily snowfall events. Thus, our analysis of bias corrected CMIP6 data^17,18^ shows substantial evidence for intensifying extreme snowfall under strong global warming (SSP5-RCP8.5).

The exclusive consideration of SSP5-RCP8.5 might constitute a limitation^28^, but in the short to medium run assuming no meaningful mitigation might be appropriate, as commented recently^29^. Moreover, slower warming in lower emissions scenarios shows similar trends of intensification of extreme snowfall events, as shown in the supplement for SSP3-RCP7.0 (Figs. S13–S17) in contrast to relatively small changes for SSP1-RCP2.6 (Figs. S18–S22).

We find increasing percentiles and expected extreme magnitude of daily snowfall for large areas until at least the middle of the 21st century. In contrast to this, mean daily snowfall decreases sharply. Analysing the observed changes weighted by population we find an increasing share of population exposed to strongly intensifying extreme snowfall events in the next decades, while the share of the population exposed to decreasing extreme snowfall grows as well, especially in the second half of the century to lead to a majority of the population being exposed to decreasing extreme snowfall percentiles at the end of the century. We also observe a regional divergence between warmer regions like Western Europe, moderate regions like the North American east coast, and snow-prone regions like Northern Asia. As an explanation for this divergence, we suggest changes in the number of days with potentially optimal temperature conditions for extreme snowfall events. These lead to increasing humidity and hence more intense extreme snowfall events. Thus, for snow-prone high-latitude regions, higher temperatures enable more extreme snowfall events until the end of the century.

The divergence between extreme events and mean daily snowfall is in line with previous studies^5,6,12^. The global circulation model data used in this study offer only a coarse resolution of $0.5^{\circ }\times 0.5^{\circ }$ compared to studies using regionalised climate models^8^, thus we are not able to conclude anything about the regional occurrence of snowstorms or similar local extreme events. Due to the bias-correction of our data we are optimistic to provide enhanced results compared to previous studies of CMIP5 data^5,6,9,10^. Our model ensemble shows good agreement on the described intensification of daily snowfall events. Thus, future research might combine the data from global climate models and regionalised climate models to fully estimate future extreme snowfall events like regional snowstorms. Finally, the introduced concept of tail risk sensitive analysis utilising the expected extreme magnitude could be applied to estimate potential impacts of extreme events in general and to the identification of optimal adaptation policies.

In summary, we show that there is substantial evidence that global warming and the resulting changes of the hydrological cycle may lead to intensification of daily snowfall extreme events in the coming decades. These changes diverge between regions and the immediate and more distant future. In particular, there is a contrast between decreasing extreme snowfall percentiles in lower latitude regions like Western Europe and higher latitude regions experiencing increasing extreme snowfall percentiles.

## Methods

### Definition of expected extreme magnitude (EEM)

We aim to improve upon exclusive consideration of the *i*th percentile $p_i$ of the random variable of daily values for a weather event *D*. To this end, we define the measure of *expected extreme magnitude (EEM)* on level *i* as the conditional expectation of a weather event *D* given that *D* exceeds ${p}_i$, the *i*th percentile of *D*:

1 $$\begin{aligned} EEM_i := \mathbb {E} \left[ D \vert D \ge {p}_i \right] , \end{aligned}$$

i.e. the mean magnitude of the events exceeding the *i*th percentile.

This conditional expectation with respect to the percentile is known in the field of financial risk management as *conditional Value at Risk*^20,21^ or *Expected Shortfall*^22^. It is commonly applied as a risk measure for the loss distribution *L* of a financial portfolio. By building on the common risk measure *Value at Risk*, the *i*th percentile of *L*, it measures the risk based on all potential losses exceeding the *i*th percentile. Due to the consideration of all realisations of the analysed random variable above the specified percentile, it is sensitive to changes of risk in the tail of the distribution.

We simplify Eq. (1) according to our application, keeping the percentile fixed as the control climate baseline percentile $\tilde{p}_i$. Thus, $\tilde{p}_i$ is deterministic and in our application expected extreme magnitude is simplified to the average of weather events $\left\{ {D_j}\right\} _{j\in 1,\dots ,N}$ that exceed the baseline *i*th percentile $\tilde{p}_i$:

2 $$\begin{aligned} EEM_i = \frac{\sum _{j \in 1,\dots ,N} \left[ D_j\vert D_j \ge \tilde{p}_i \right] }{N}, \end{aligned}$$

where N is the number of all considered realisations of *D*.

This enables a comparison of the expected magnitude of extreme events under future climate scenarios to the expected magnitude of the historical baseline scenario. Since in contrast to a financial portfolio, we are not able to influence the distribution of weather events *D* by reallocating assets, the baseline percentile constitutes a valid threshold also for future time periods.

This simplification is not without caveats, since the probabilistic interpretation of the events exceeding the *i*th percentile of the analysed period is replaced by a comparison with the events exceeding the *i*th percentile of the (historical) baseline. Nonetheless, since preparation against extreme snowfall events is based on historical experiences, we strongly favor the fixed baseline percentile approach to the changing percentile threshold as it would be used for conditional Value at Risk due to the following arguments. As can be seen in Fig. S6, using the changing future percentiles as a baseline, i.e. just as for the original conditional Value at Risk, yields a measure which tracks changes in the percentile quite closely. Our definition of EEM enables a complementary analysis adding important insights about the most extreme snowfall events. Even if they become rarer as follows from decreasing percentiles, the remaining events are intensifying and thus EEM uncovers important information that would be lost using the changing percentile as a threshold. Moreover, for increasing percentiles our definition of EEM yields conservative estimates of intensification.

In future applications of this measure, an advanced modelling of the distribution of daily weather extremes *D* might enable an application of Eq. (1) for the management of extreme weather risks. For example, adaptation policies could be optimised by modelling the expected impacts of weather extremes after implementation of these policies and adaptation priorities could be identified following the maximisation of the expected reduction of extreme damages. There remain substantial challenges to this approach, since caution is required to model the relevant tails of the distributions accurately and the numerical simulation of conditional expectations is challenging due to their path-dependency, rendering standard Monte-Carlo methods computationally infeasible.

### Calculation of daily snowfall from climate model output

To ensure a robust measurement of daily snowfall per grid cell, we have applied the following simple transformation to model output precipitation (*pr*) based on surface temperature (*tas*). Snowfall (*prsn*) is assumed to occur if and only if the surface temperature is below 0 ${^\circ }$C, i.e.

3 $$\begin{aligned} prsn :={ \mathbb{1}}_{tas\le 0^{\circ } C} pr, \end{aligned}$$

as also commonly used by hydrological models.

#### Auxiliary data used

For population weighted analysis, all grid cell data are weighted by 2020 population data^30^ to show trends in impacts on human activities. The weights are scaled to the total population of the analysed area. This introduces a limitation, because we do not consider population development for the historical data and under different scenarios, e.g. SSPs. Thus, our analysis focuses on the changes of extreme snowfall without considering population shifts.

For elevation data, we use data from the global land data assimilation project^31^.

#### Calculation of snowfall statistics

All statistics (mean, percentiles, expected extreme magnitude) are calculated per grid cell, using the $0.5^{\circ }$ resolution of the ISIMIP data^17–19^, for $T=10$ year windows of the analysed time frames. All percentiles are estimated based on standard percentile estimation techniques implemented in scipy^32^. The analysis was facilitated by the IRIS python package^33^.

Area averages are calculated from ocean-masked data, weighting the grid cell data by area with weights given by

4 $$\begin{aligned} r^2\left( lon_1-lon_0\right) \left( sin\left( lat_1\right) -sin\left( lat_0\right) \right) , \end{aligned}$$

where *r* denotes the radius of the earth, approximated as 6, 367, 470 m^33^.

The baseline values are calculated as an average of decadal data from the baseline period of 1851–1920. These are used as basic values for all baseline relative percentages.

## Supplementary Information

Supplementary Information 1.

## Data Availability

All code used for analysis and data that support the findings of this study are available from the corresponding author upon request.
