# Supplementary material for: Regions of intensification of extreme snowfall under future warming
Source: Sci Rep. 2021 Aug 17;11:16621. doi: 10.1038/s41598-021-95979-4 (PMC8371008; doi:10.1038/s41598-021-95979-4)
Supplement: Supplementary file 1 — Supplementary Information 1. [file 41598_2021_95979_MOESM1_ESM.pdf]

Supplementary information

Regions of intensification of extreme snowfall under future warming

Lennart Quante [a,b], Sven N. Willner [a,*], Robin Middelanis [a,b], Anders Levermann[a,b,c]

[a] Potsdam Institute for Climate Impact Research, Telegrafenberg A56, Potsdam, Germany

[b] Potsdam University, Karl-Liebknecht Str. 24, Potsdam, Germany

[c] Columbia University, LDEO, Palisades, New York, USA

Fig. S1: Regions considered for localised analysis

Tbl. S1: Overview of ensemble models.

Fig. S2: 99th percentile: Changes of daily snowfall metrics relative to historical baseline (1851–1920).

Fig. S3: High-model agreement on mean daily snowfall changes.

Fig. S4: High-model agreement on 99.9th percentile changes.

Fig. S5: Mixed model agreement on EEM above the 99.9th baseline percentile changes.

Fig. S6: Contrasting global trends of mean daily snowfall and extreme snowfall measures with alternative definition of EEM — using the current percentile as a threshold.

Fig. S7: Maps of extreme statistics for CMIP model MPI-ESM1-2-HR without bias correction

Fig. S8: Maps of mean for CMIP model MPI-ESM1-2-HR without bias correction

Fig. S9 Global snowfall trends for CMIP model MPI-ESM1-2-HR without bias correction

Fig. S10 Regional snowfall statistics for CMIP model MPI-ESM1-2-HR without bias correction

Fig. S11 Regional temperature trends for CMIP model MPI-ESM1-2-HR without bias correction

Fig. S12: SSP5-RCP8.5: Area weighted statistics of change bins for 99.9th percentile.

Fig. S13: Maps of extreme statistics for SSP3-RCP7.0

Fig. S14: Maps of mean for SSP3-RCP7.0

Fig. S15 Global snowfall trends for SSP3-RCP7.0

Fig. S16 Regional snowfall statistics for SSP3-RCP7.0

Fig. S17 Regional temperature trends for SSP3-RCP7.0

Fig. S18: Maps of extreme statistics for SSP1-RCP2.6

Fig. S19: Maps of mean for SSP1-RCP2.6

Fig. S20 Global snowfall trends for SSP1-RCP2.6

Fig. S21: Regional snowfall statistics for SSP1-RCP2.6

Fig. S22 Regional temperature trends for SSP1-RCP2.6

Fig. S23: Regional trends of temperatures below freezing point

Fig. S24: Global trends regarding temperature bins

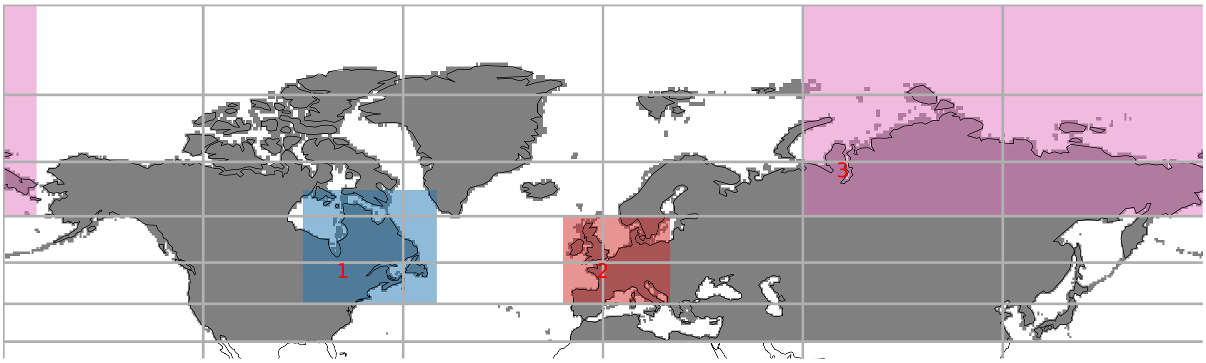

Figure S1: Regions considered for localised analysis. Only land area is Map created using the cartopy 0.17[24] library based on GSHHG shapes[25].

**Climate model**
CanESM5
CNRM-CM6-1
CNRM-ESM2-1
EC-Earth3
GFDL-ESM4
IPSL-CM6A-LR
MIROC6
MPI-ESM1-2-HR
MRI-ESM2-0
UKESM1-0-LL

Table S1: Climate models used in the ensemble

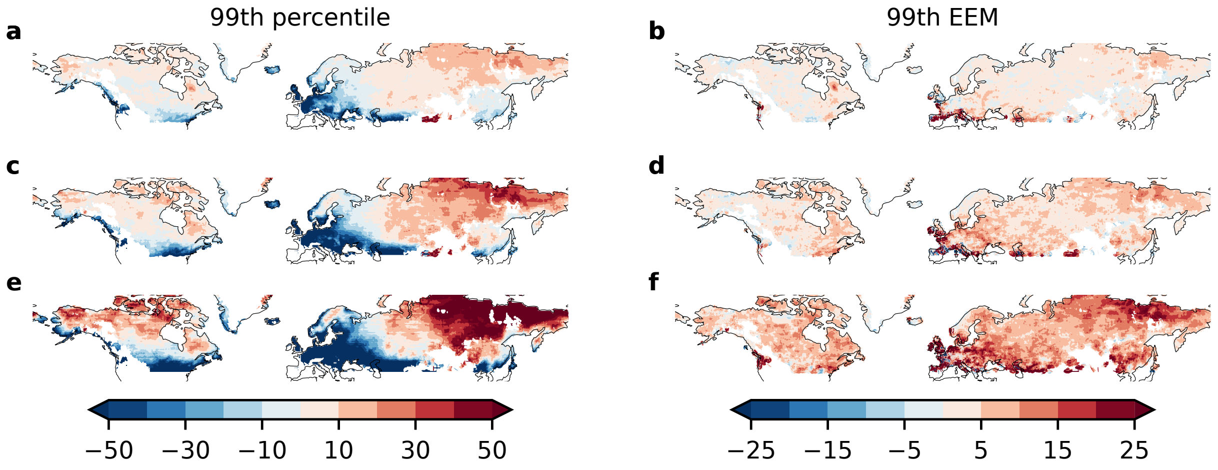

Figure S2: 99th percentile: Changes of daily snowfall metrics relative to historical baseline (1851–1920). Percentage change of **a,c,e** 99th percentile and **b,d,f** 99th expected extreme magnitude. **a,b** 2021–2030, **c,d** 2051–2060, **e,f** 2091–2100. Maps created using the cartopy 0.17[24] library based on GSHHG shapes[25].

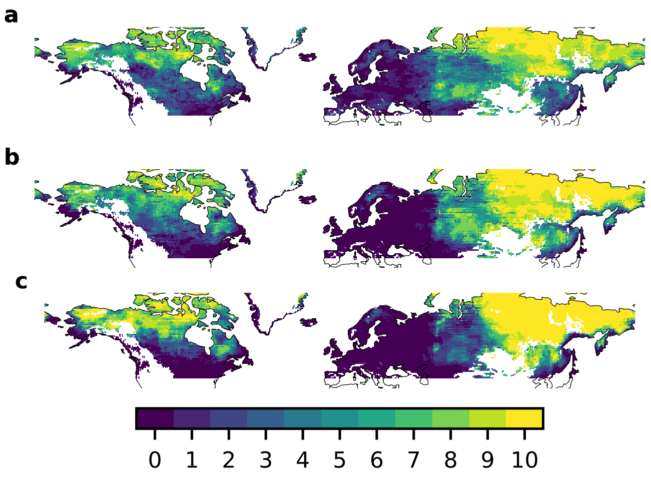

Figure S3: High-model agreement on mean daily snowfall changes. Number of models showing an increasing mean daily snowfall; **a** 2021–2030, **b** 2051–2060, **c** 2091–2100. Maps created using the cartopy 0.17[24] library based on GSHHG shapes[25].

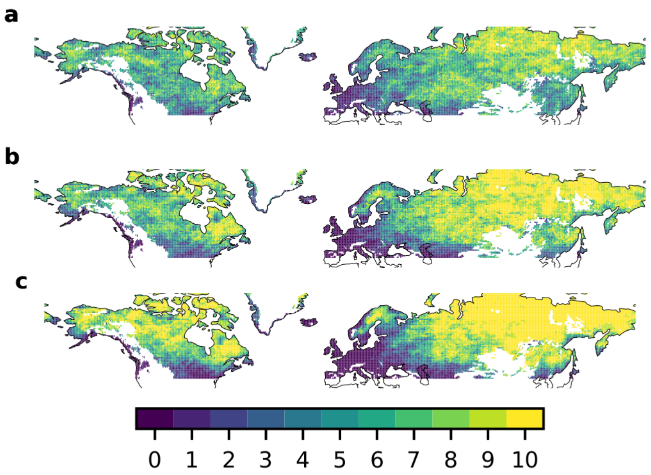

Figure S4: High-model agreement on 99.9th percentile changes. Number of models showing an increasing 99.9th percentile; **a** 2021–2030, **b** 2051–2060, **c** 2091–2100. Maps created using the cartopy 0.17[24] library based on GSHHG shapes[25].

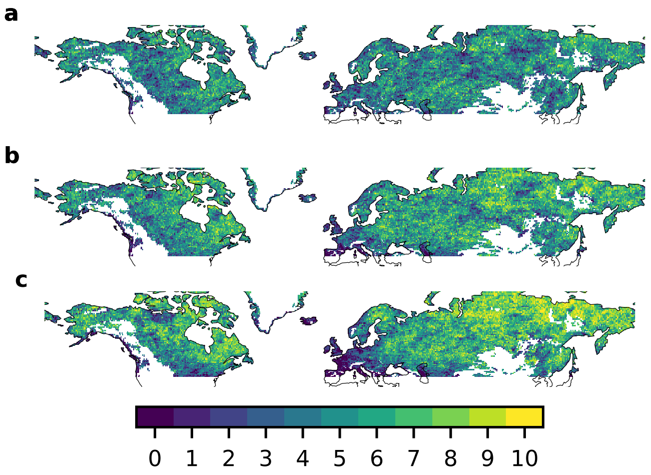

Figure S5: Mixed model agreement on EEM above the 99.9th baseline percentile changes. Number of models showing an increasing 99.9th EEM; **a** 2021–2030, **b** 2051–2060, **c** 2091–2100. Maps created using the cartopy 0.17[24] library based on GSHHG shapes[25].

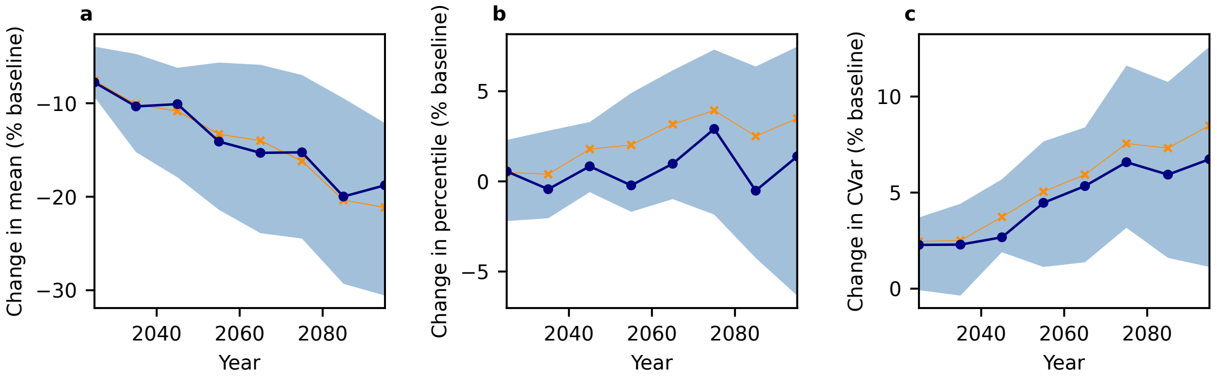

Figure S6: Contrasting global trends of mean daily snowfall and extreme snowfall measures — using the current percentile as a threshold (as in Fig. 3 with CVar instead of EEM, decadal statistics, Northern Hemisphere north of 40°N, for SSP5-RCP8.5). All values are area-weighted and relative to the baseline (1851–1920) climate. **a** Mean, **b** 99.9th percentile, **c** Conditional Value at Risk of the 99.9th percentile. Blue line shows the model ensemble median, shaded areas denote the likely range (16.7th to 83.3rd percentiles). Orange line shows statistics for all ten models combined into one time series ensemble.

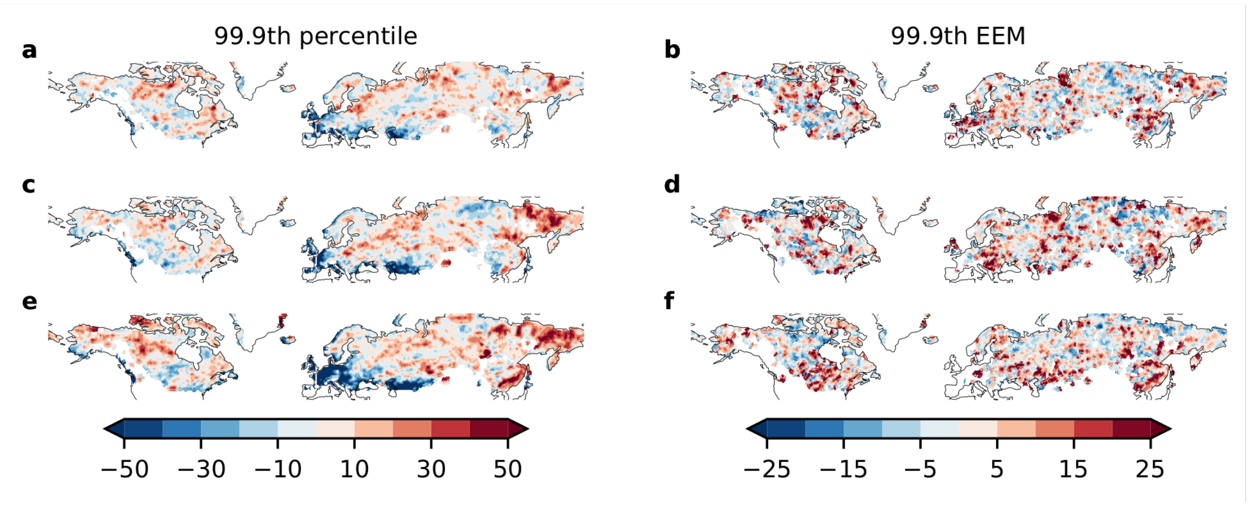

Figure S7: Fig. 1 for CMIP MPI-ESM1-2-HR model without bias correction, SSP5-RCP8.5: Changes of daily snowfall metrics relative to historical baseline (1851–1920). Percentage change of **a,c,e** 99.9th percentile and **b,d,f** 99.9th expected extreme magnitude. **a,b** 2021–2030, **c,d** 2051–2060, **e,f** 2091–2100. Maps created using the cartopy 0.17[24] library based on GSHHG shapes[25].

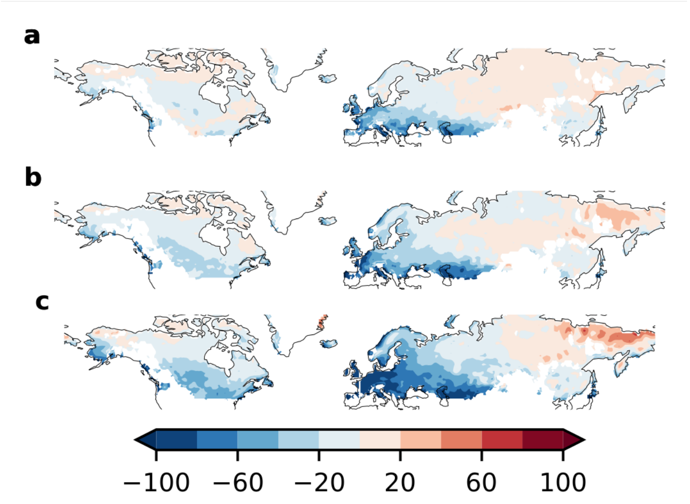

Figure S8: Fig. 2 for CMIP MPI-ESM1-2-HR model without bias correction, SSP5-8.5: Changes of daily snowfall mean relative to historical baseline (1851–1920). **a** 2021–2030, **b** 2051–2060, **c** 2091–2100. Maps created using the cartopy 0.17[24] library based on GSHHG shapes[25].

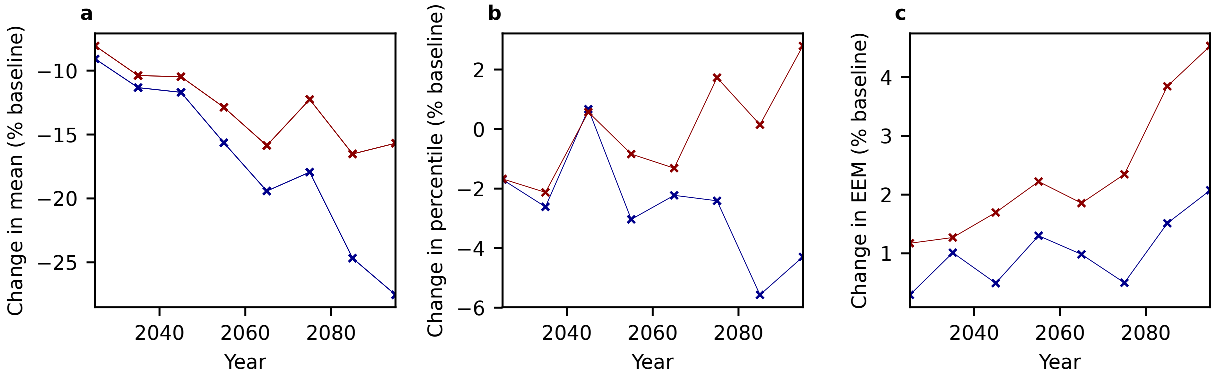

Figure S9: Fig. 3 for CMIP without bias correction –blue line– and ISIMIP bias corrected –red line– MPI-ESM1-2-HR model, SSP5-8.5: Contrasting global trends of mean daily snowfall and extreme snowfall measures (elevation below 1000m, decadal statistics, Northern Hemisphere north of 40°N). All values are relative to the baseline (1851–1920) climate. **a** Mean, **b** 99.9th percentile, **c** Expected extreme magnitude above the 99.9th baseline percentile.

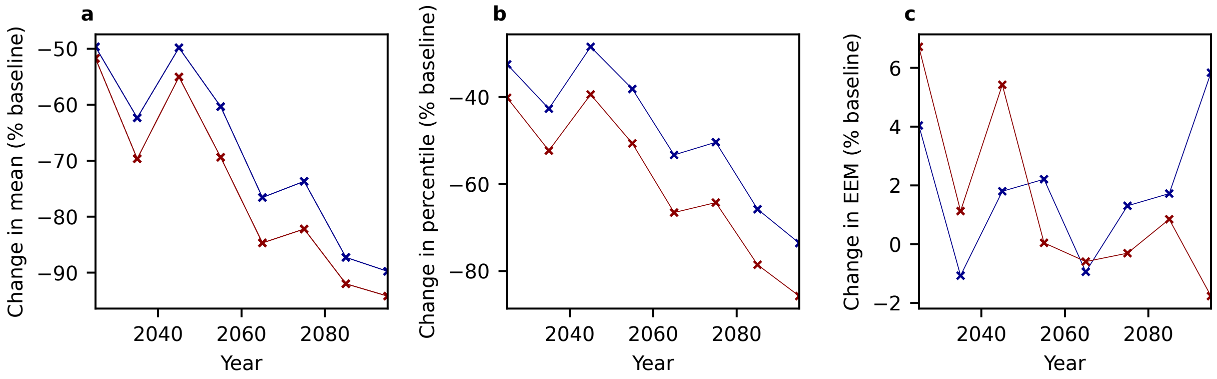

S10.1. Western Europe

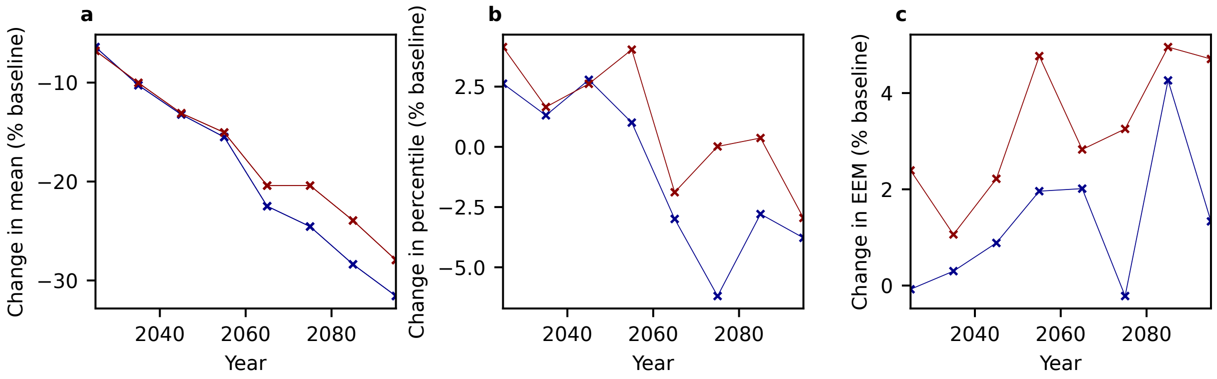

S10.2. North America east coast

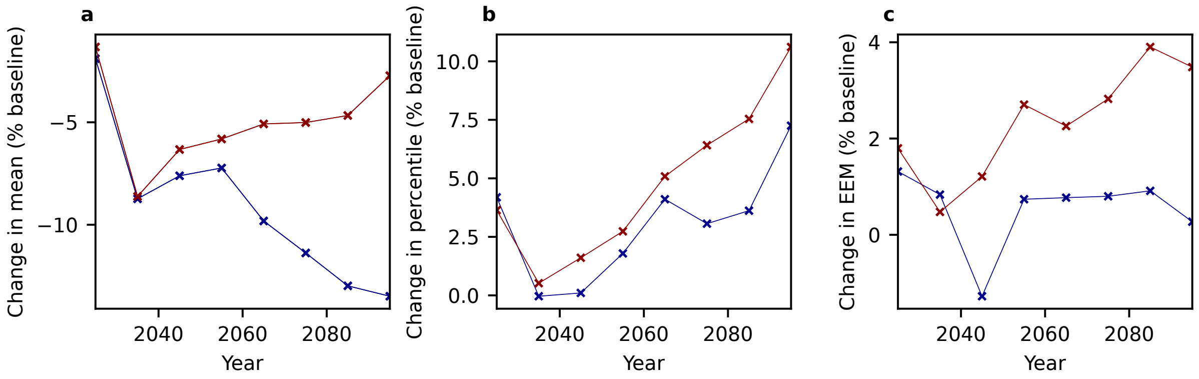

S10.3. Northern Asia

Figure S10: Fig. 5 for CMIP without bias correction –blue line– and ISIMIP bias corrected –red line– MPI-ESM1-2-HR model: Regional differences of changes in daily snowfall statistics (elevation below 1000m, decadal statistics, SSP5-RCP8.5). All values are relative to the baseline (1851–1920) climate. **a** Mean, **b** 99.9th percentile, **c** Expected extreme magnitude above the 99.9th baseline percentile.

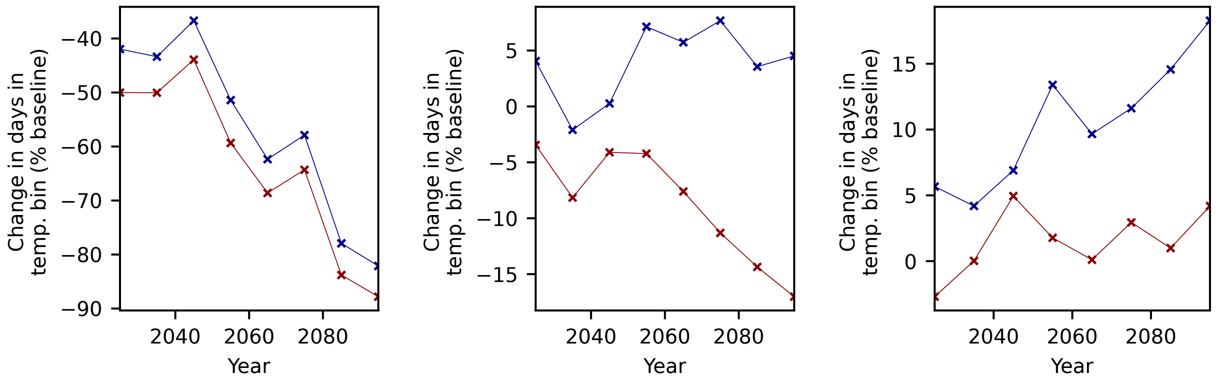

S11.1. Western Europe **S11.2. North America east coast** **S11.3. Northern Asia**

Figure S11: Fig. 6 for CMIP without bias correction –blue line– MPI-ESM1-2-HR model and ISIMIP bias corrected –red line– MPI-ESM1-2-HR model, SSP5-8.5: Regional differences in trend of days with surface temperature between −2.5℃ and −1.5℃. For elevation below 1000m, decadal statistics. All values are relative to the baseline (1851–1920) climate.

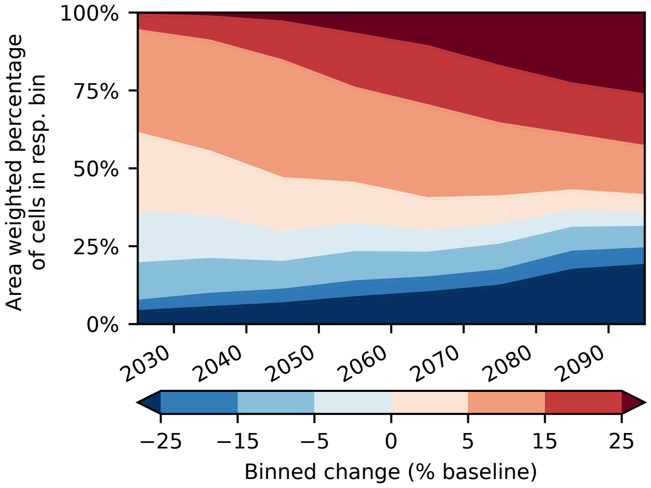

Figure S12: Area exposed to strongly (more than 5 percentage points compared to baseline levels) intensifying snowfall events grows almost until the end of the century - Global area weighted trend of 99.9th percentile (elevation below 1000m, decadal statistics, Northern Hemisphere north of 40°N, SSP5-RCP8.5). Binned according to change relative to the baseline (1851–1920) climate. Coloured area represents the area weighted percentage of cells in the respective bin.

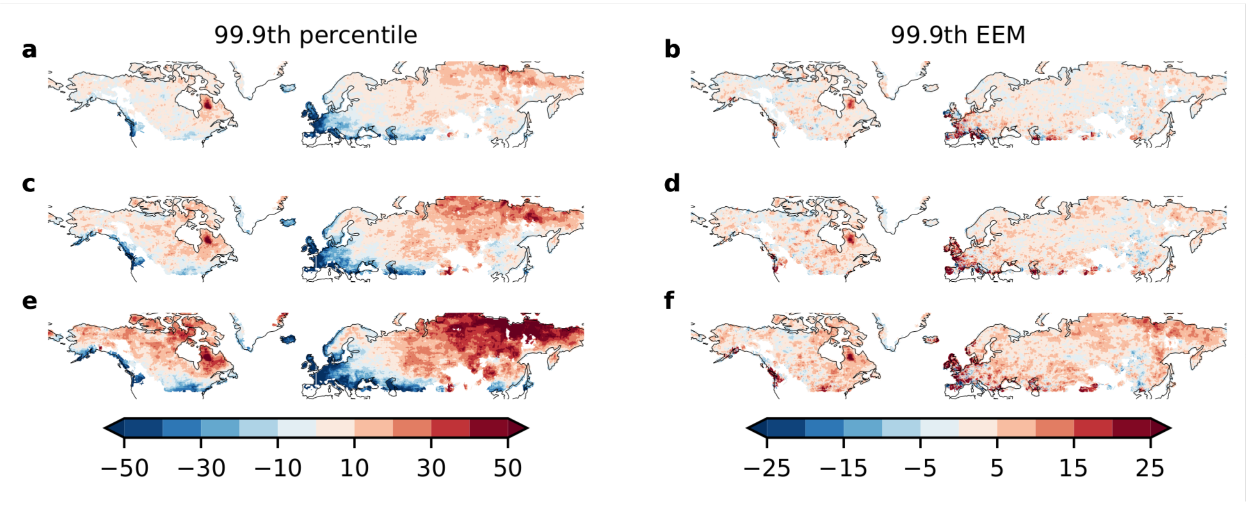

Figure S13: SSP3-RCP7.0: Changes of daily snowfall metrics relative to historical baseline (1851–1920). Percentage change of **a,c,e** 99.9th percentile and **b,d,f** 99.9th expected extreme magnitude. **a,b** 2021–2030, **c,d** 2051–2060, **e,f** 2091–2100. Maps created using the cartopy 0.17[24] library based on GSHHG shapes[25].

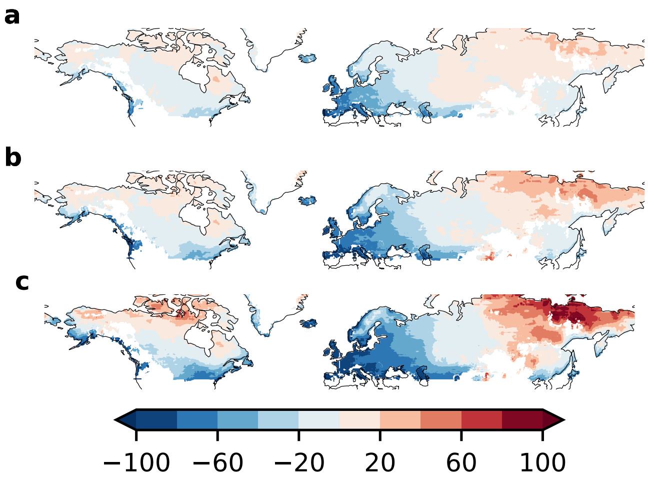

Figure S14: SSP3-RCP7.0: Changes of daily snowfall mean relative to historical baseline (1851–1920). **a** 2021–2030, **b** 2051–2060, **c** 2091–2100. Maps created using the cartopy 0.17[24] library based on GSHHG shapes[25].

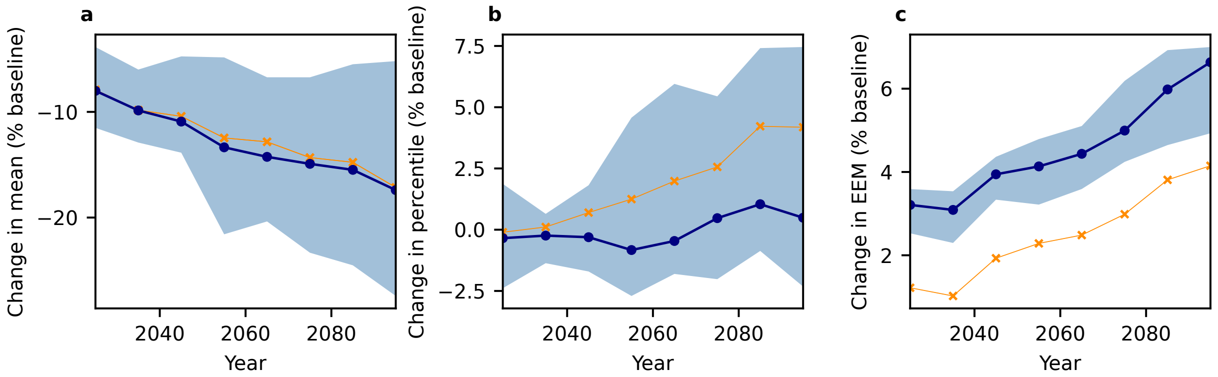

Figure S15: SSP3-RCP7.0: Contrasting global trends of mean daily snowfall and extreme snowfall measures (elevation below 1000m, decadal statistics, Northern Hemisphere north of 40°N). All values are relative to the baseline (1851–1920) climate. **a** Mean, **b** 99.9th percentile, **c** Expected extreme magnitude above the 99.9th baseline percentile. Blue line shows the model ensemble median, shaded areas denote the likely range (16.7th to 83.3rd percentiles). Orange line shows statistics for all ten models combined into one time series ensemble.

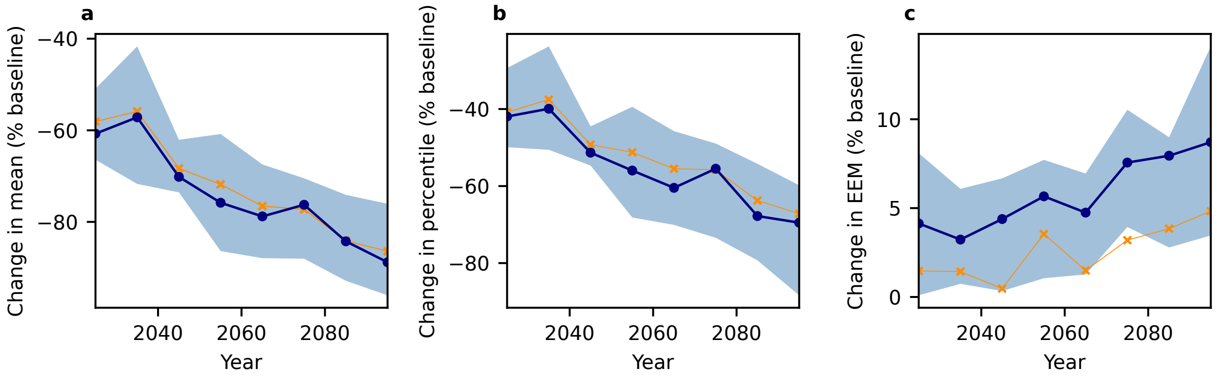

S16.1. Western Europe

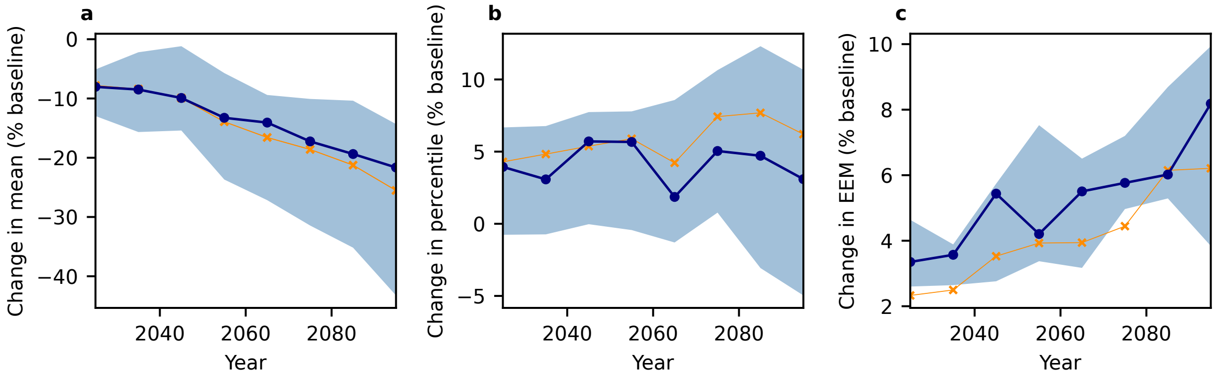

S16.2. North America east coast

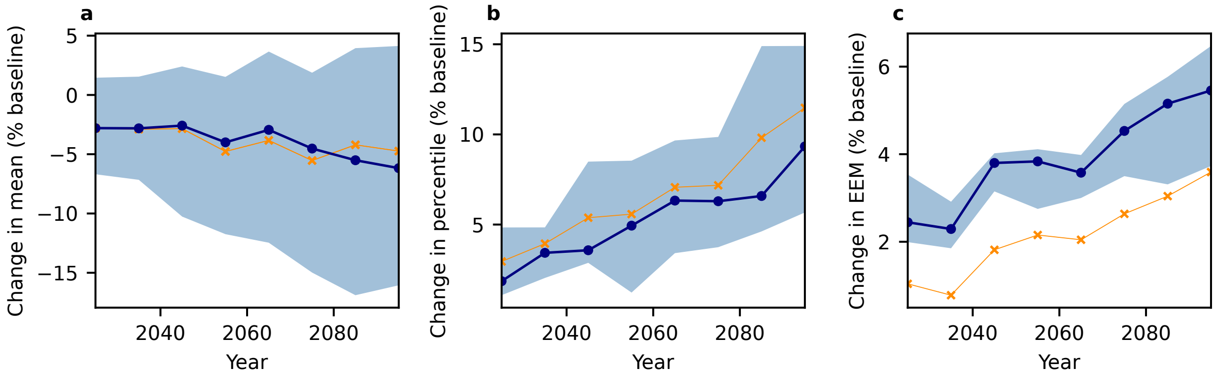

S16.3. Northern Asia

Figure S16: SSP3-RCP7.0: Regional differences of changes in daily snowfall statistics (elevation below 1000m, decadal statistics, SSP5-RCP8.5). All values are relative to the baseline (1851–1920) climate. **a** Mean, **b** 99.9th percentile, **c** Expected extreme magnitude above the 99.9th baseline percentile. Blue line shows the model ensemble median, shaded areas denote the likely range (16.7th to 83.3rd percentiles). Orange line shows statistics for all ten models combined into one time series ensemble.

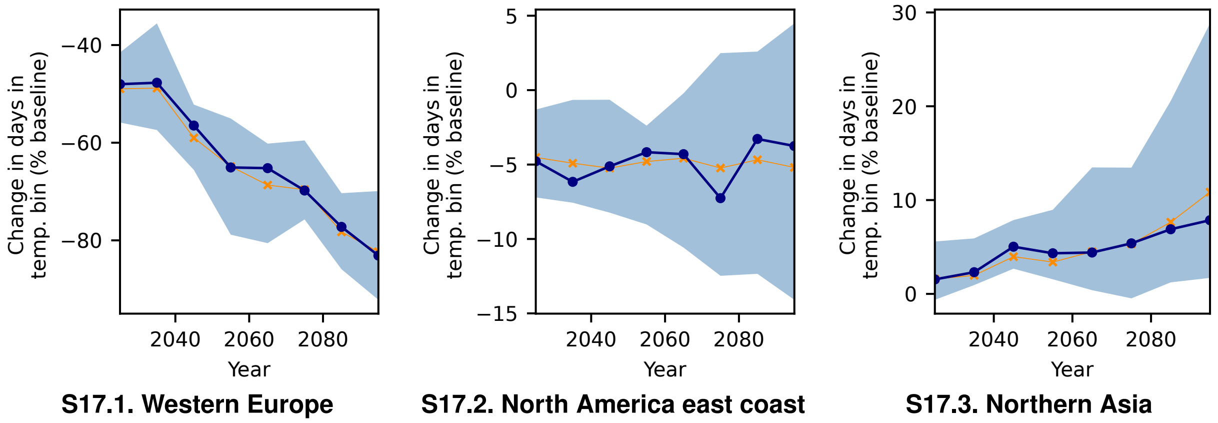

Figure S17: SSP3-RCP7.0: Regional differences in trend of days with surface temperature between -2.5℃ and -1.5℃. For elevation below 1000m, decadal statistics. All values are relative to the baseline (1851–1920) climate. Blue line shows the model ensemble median, shaded areas denote the likely range (16.7th to 83.3rd percentiles). Orange line shows statistics for all ten models combined into one time series ensemble.

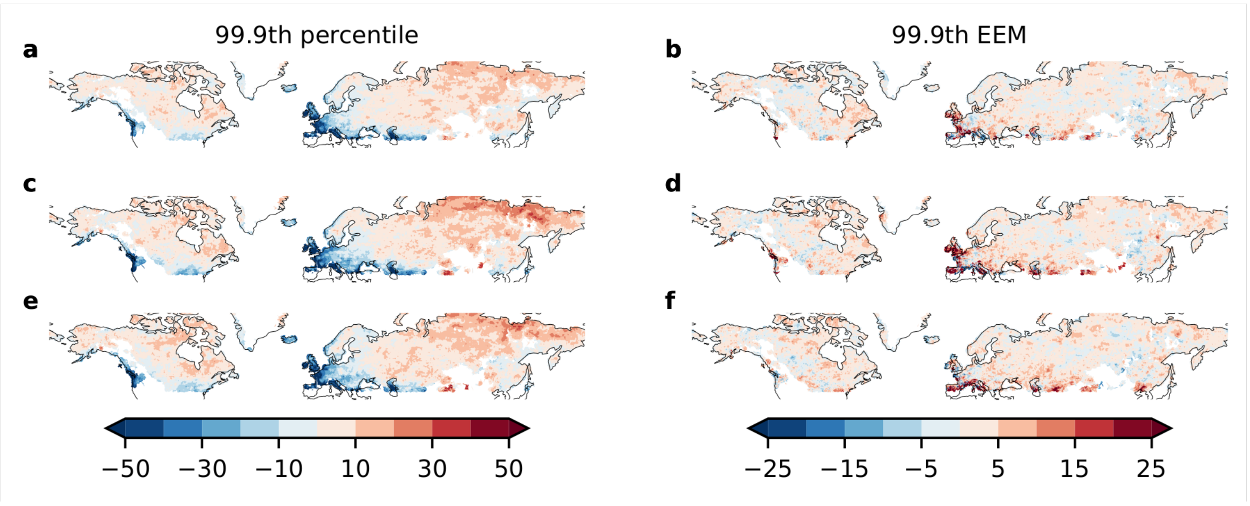

Figure S18: SSP1-RCP2.6: Changes of daily snowfall metrics relative to historical baseline (1851–1920). Percentage change of **a,c,e** 99.9th percentile and **b,d,f** 99.9th expected extreme magnitude. **a,b** 2021–2030, **c,d** 2051–2060, **e,f** 2091–2100. Maps created using the cartopy 0.17[24] library based on GSHHG shapes[25].

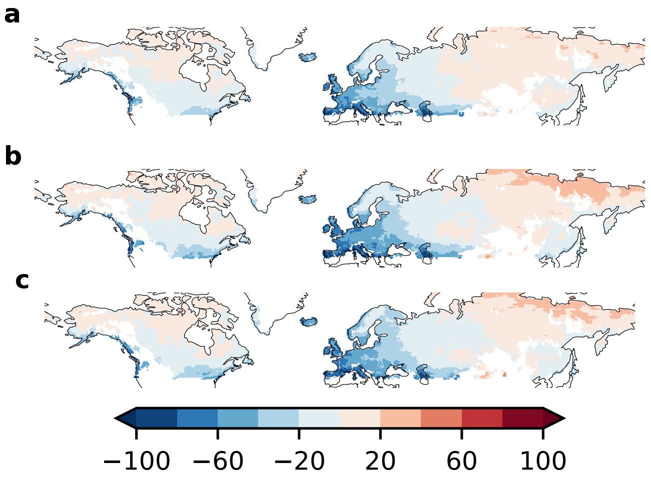

Figure S19: SSP1-RCP2.6: Changes of daily snowfall mean relative to historical baseline (1851–1920). **a** 2021–2030, **b** 2051–2060, **c** 2091–2100. Maps created using the cartopy 0.17[24] library based on GSHHG shapes[25].

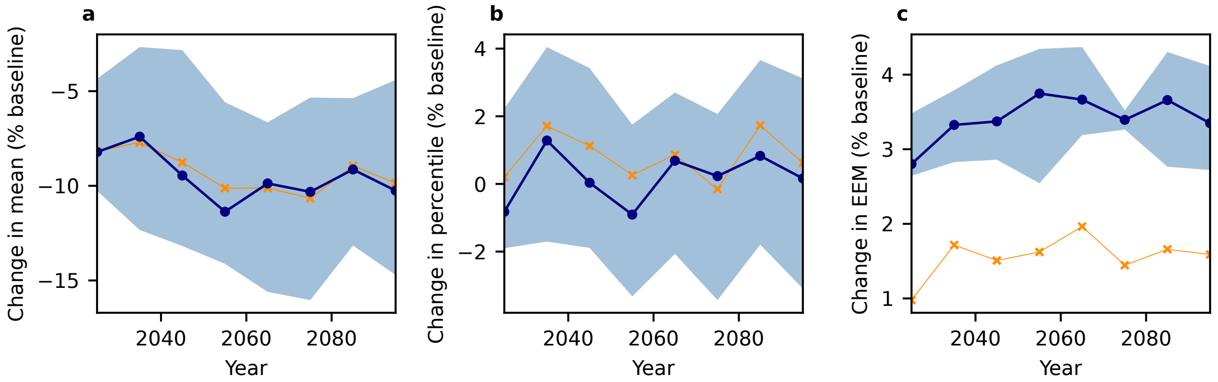

Figure S20: SSP1-RCP2.6: Global trends of mean daily snowfall and extreme snowfall measures (elevation below 1000m, decadal statistics, Northern Hemisphere north of 40°N). All values are relative to the baseline (1851–1920) climate **a** Mean **b** 99.9th percentile **c** Expected extreme magnitude above the 99.9th baseline percentile. Blue line shows the model ensemble median, shaded areas denote the likely range (16.7th to 83.3rd percentiles). Orange line shows statistics for all ten models combined into one time series ensemble.

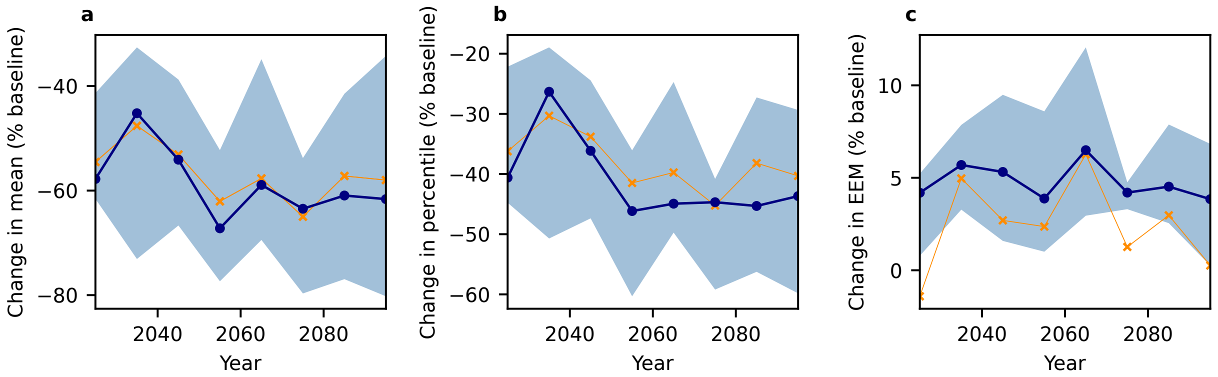

S21.1. Western Europe

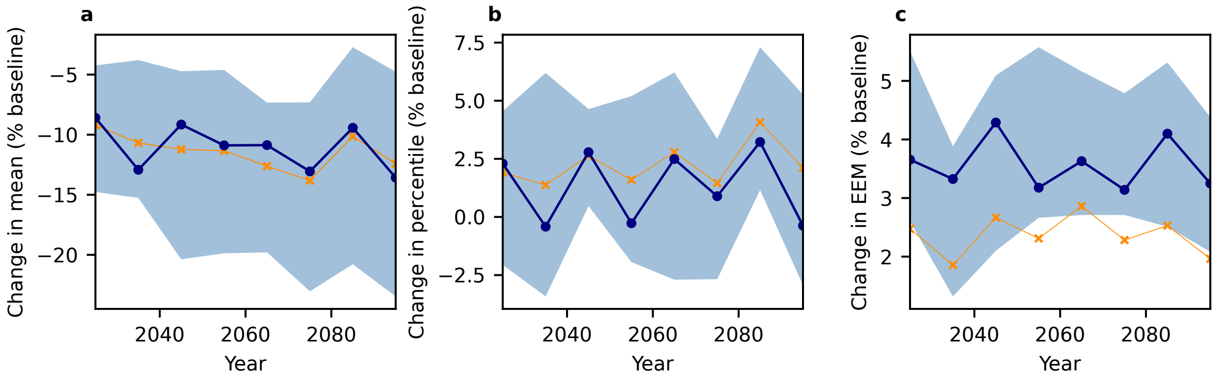

S21.2. North America east coast

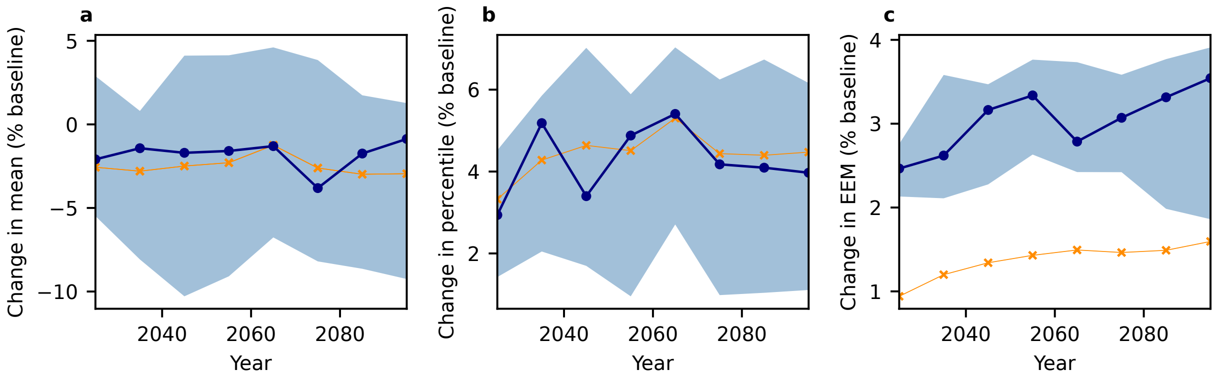

S21.3. Northern Asia

Figure S21: SSP1-RCP2.6: Regional differences of changes in daily snowfall statistics (elevation below 1000m, decadal statistics, SSP5-RCP8.5). All values are relative to the baseline (1851–1920) climate. **a** Mean, **b** 99.9th percentile, **c** Expected extreme magnitude above the 99.9th baseline percentile. Blue line shows the model ensemble median, shaded areas denote the likely range (16.7th to 83.3rd percentiles). Orange line shows statistics for all ten models combined into one time series ensemble.

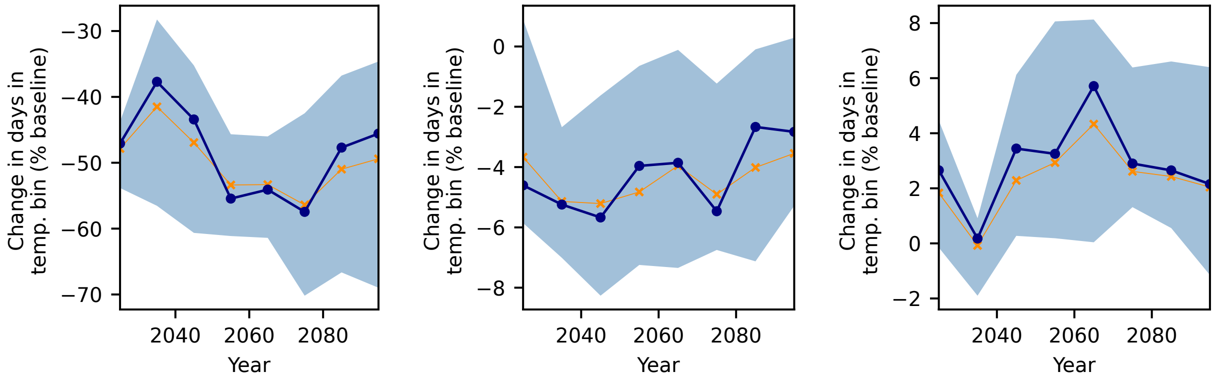

S22.1. Western Europe **S22.2. North America east coast** **S22.3. Northern Asia**

Figure S22: SSP1-RCP2.6: Regional differences in trend of days with surface temperature between −2.5℃ and −1.5℃ (elevation below 1000m, decadal statistics). All values are relative to the baseline (1851–1920) climate. Blue line shows the model ensemble median, shaded areas denote the likely range (16.7th to 83.3rd percentiles). Orange line shows statistics for all ten models combined into one time series ensemble.

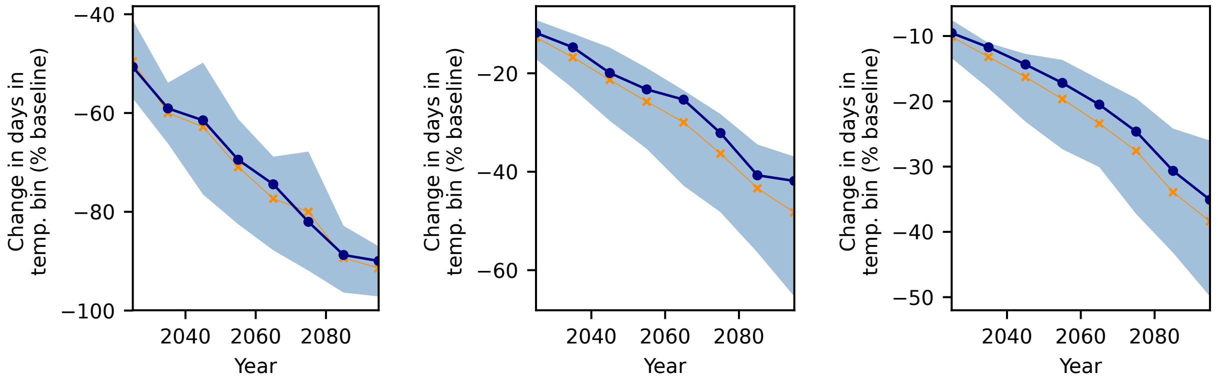

S23.1. Western Europe **S23.2. North America east coast** **S23.3. Northern Asia**

Figure S23: Days with surface temperature below 0 ℃ (elevation below 1000m, decadal statistics, comparison between different regions, SSP5-RCP8.5). All values are relative to the baseline (1851–1920) climate. Blue line shows the model ensemble median, shaded areas denote the likely range (16.7th to 83.3rd percentiles). Orange line shows statistics for all ten models combined into one time series ensemble.

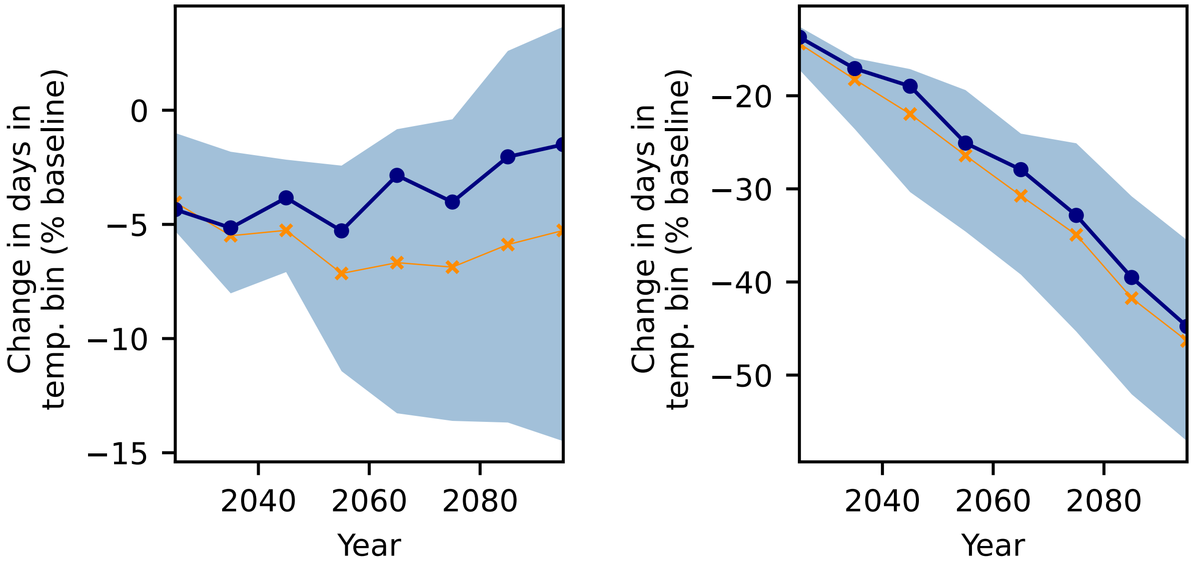

S24.1. Temp. in [-2.5°C, -1.5°C] **S24.2. Temp. below 0°C**

Figure S24: Comparison between different temperature bins. Decadal statistics of days with surface temperature in given temperature range for elevation below 1000m for the Northern Hemisphere north of 40°N for SSP5-RCP8.5. All values are relative to the baseline (1851–1920) climate.
